# Supernumerary Tusks in African Savanna Elephants (*Loxodonta africana*): A Case Series and Consideration of Developmental Mechanisms

**DOI:** 10.3390/ani16192972

**Published:** 2026-09-22

**Authors:** Gerhard Steenkamp, Sonja Catharina Boy

**Affiliations:** 1 Greens Zoological Rescue and Rehabilitation Centre, Vantara, Jamnagar 361142, Gujarat, India; 2 Department of Companion Animal Clinical Studies, Faculty of Veterinary Science, University of Pretoria, Private Bag X04, Onderstepoort 0110, South Africa; 3 Lancet Laboratories, Pencardia Building, 509 Pretorius Street, Arcadia, Pretoria 0083, South Africa; sonja.iaop@gmail.com

**Keywords:** elephant, tusk, supernumerary teeth, hyperdontia, elodont dentition

## Abstract

Supernumerary teeth are well recognised in humans and domestic animals but are rarely reported in elephants. This case series describes four African savanna elephants (*Loxodonta africana*) with multiple tusk structures occupying a single alveolus. A 7-year-old bull presented with a deformed left tusk and behavioural changes. Clinical examination revealed two coronal openings, followed by eruption of three accessory tusks. Conservative intracavitary treatment and selective surgical removal of the accessory tusks preserved the primary tusk, which maintained normal eruption and function at 36-month follow-up. Additional cases comprised a bull that developed three tusks after traumatic fracture and removal of the fractured tusk remnant, an adult female with two tusks in one alveolus, and a dry specimen composed of five tusks, each with a distinct pulp canal. The continued eruption, organised morphology, separate pulp canals, and surgical separability of these structures support their diagnosis as supernumerary tusks. The developmental mechanism remains uncertain, but lifelong odontogenic activity in elephant tusks may permit formation of independent dentine-producing structures, and trauma may contribute in some cases. Selective removal of accessory tusks while preserving the primary tusk can provide a favourable long-term outcome.

## Simple Summary

Elephant tusks are continuously growing upper incisor teeth. Rare historical accounts describe elephants with more than one tusk growing from the same socket, but the condition has not been documented in detail. We describe four African savanna elephants with two to five tusks occupying a single socket. In one young bull, three smaller tusks developed alongside the main tusk. The smaller tusks were surgically removed while the main tusk was preserved. It continued to grow normally and remained functional three years later. A second bull developed three tusks after a severe tusk fracture, suggesting that trauma may contribute in some cases. The other cases involved an adult female with two tusks in one socket and a dry specimen composed of five tusks. The separate pulp canals, continued growth, and surgical separability of these structures support their diagnosis as supernumerary tusks, although their developmental origin remains uncertain. Recognising this condition may help veterinarians distinguish it from tumours or other tusk abnormalities and may support conservative treatment aimed at preserving the main tusk.

## 1. Introduction

African savanna elephant (*Loxodonta africana*) dental formulae are commonly expressed as I1/0 C0/0 PM3/3 M3/3 [1]. Several systems of nomenclature have been proposed for Elephantidae cheek teeth. In this manuscript, the six sequential cheek teeth of African elephants (*Loxodonta africana*) are designated M1–M6 following Laws (1966) [2]. The tusks represent the maxillary second incisor teeth [1]. They are continuously growing teeth that do not undergo normal root closure and retain lifelong odontogenic activity. Such teeth have traditionally been classified as hypselodont in comparative anatomical literature [3,4], although Kertesz [5] subsequently proposed the term elodont to emphasise continuous growth rather than crown morphology. During odontogenesis, reciprocal epithelial–mesenchymal interactions initiate differentiation of ameloblasts and odontoblasts [6]. Following eruption, the enamel cap of the elephant tusk is rapidly lost, while dentine production by odontoblasts lining the pulp cavity continues throughout life [1].

Developmental abnormalities of elephant dentition are uncommon. Hypodontia and tusklessness, particularly in African savanna elephants, are well documented and appear to have a heritable basis, possibly linked to X-chromosomal inheritance [7,8]. Reports of multiple tusk structures in elephants are rare and are largely confined to historical observations, museum specimens, or anecdotal field reports. Historical accounts have described elephants possessing three, four, five, seven, or even nine tusks, although these observations generally lacked clinical documentation or pathological evaluation [1,9]. Colyer described several examples of multiple tusks and proposed that at least some cases may have been associated with trauma to persistently growing tusks [10]. Despite these historical reports, the clinical presentation, biological behaviour, and developmental origin of supernumerary tusks remain poorly understood. It appears that Junge (1941) was the first to describe supernumerary tusks in a Sumatran elephant [11].

Hyperdontia refers to the presence of more than the normal number of teeth, and the additional teeth are termed supernumerary teeth [12]. Several theories have been proposed regarding the development of supernumerary teeth in humans, including phylogenetic reversion (atavism) [13], splitting of a developing tooth germ (dichotomy theory) [14], localised hyperactivity of the dental lamina [15], hereditary influences [16], and trauma [17]. However, the applicability of these mechanisms to continuously growing elodont teeth such as elephant tusks remains uncertain.

The term “tusklet”, apparently introduced by Sikes [18], has been used to describe smaller tusk-like structures associated with a primary tusk within the same alveolus [18,19]. To date, these structures have not been described in a detailed clinical context, and their developmental origin remains poorly understood.

The aim of this study was to describe four African savanna elephants with multiple tusk structures within a single alveolus, including one case managed surgically with long-term follow-up, and to propose possible developmental mechanisms for supernumerary tusk formation in elodont dentition. The authors prefer the term elodont, which correctly describes the biology of elephant tusks, and will favour this term throughout the paper.

## 2. Materials and Methods

### 2.1. Origin of Cases

Cases were identified opportunistically during clinical evaluation of African savanna elephants. All four cases involved unrelated elephants. Cases 1–3 were maintained in captivity. Cases 1 and 2 had been brought into captivity, whereas the origin of Case 3 was unknown. The origin and original setting of the animal represented by the dry specimen in Case 4 was not known. Specific institutional locations are not reported to respect requests for confidentiality.

### 2.2. Clinical and Anatomical Assessment

Clinical assessment was based on direct oral examination, serial photographic documentation, veterinary records and follow-up information provided by attending veterinarians and animal care staff. For the purposes of this study, the primary tusk was defined as the normally expected single maxillary second incisor tooth and, where multiple tusks were present, the largest tusk within the alveolus; all additional tusks within the same alveolus were classified as supernumerary tusks. Individual tusks were assessed by direct inspection and, where clinically accessible, manipulation. Where internal anatomy was exposed during clinical treatment or visible in the anatomical specimen, pulp canals and/or pulp tissue were assessed directly. No specimens were sectioned specifically for research purposes, and no histological or microscopic examination was carried out.

In Case 1, surgical intervention was performed under general anaesthesia using standard immobilisation protocols as part of routine veterinary management [18]. Conventional radiography of the affected tusk was attempted in this case but was non-diagnostic because of the dimensions and radiographic density of the tusk and surrounding alveolar bone.

This study comprised retrospective clinical observations and case documentation. All procedures were performed as part of routine veterinary management and welfare-related intervention; no procedure was undertaken for research purposes.

Generative artificial intelligence was used during manuscript preparation to assist with language refinement, structural organisation, and consideration of alternative wording and interpretations. It was not used to generate or analyse clinical data. All output was critically reviewed and revised by the authors.

## 3. Case Descriptions

The series comprised three clinical cases and one anatomical specimen evaluated retrospectively.

### 3.1. Case 1

A 7-year-old African elephant bull presented with an abnormal left tusk and recent behavioural changes, including reduced responsiveness to commands. Under general anaesthesia using etorphine and azaperone [20], clinical examination revealed a deformed left tusk with a flattened coronal surface and two distinct openings, approximately 12 mm and 8 mm in diameter. No haemorrhage, purulent material, or visible pulp tissue was observed. The contralateral tusk was of normal shape and size for the elephant. No previous dental trauma was recorded.

Radiographic examination was attempted but was non-diagnostic because of the density of the tusk and surrounding alveolar bone. The cavities were irrigated with sterile saline (Sodium chloride 5%, Fresenius Kabi, Halfway House, Midrand, South Africa) and dressed with calcium hydroxide powder (Slaked lime, The Dental Warehouse, Midrand, South Africa) to reduce bacterial contamination and promote tertiary dentine formation. The openings were temporarily sealed with epoxy resin (Steel quickset, Pratley, Krugersdorp, South Africa) to limit contamination until reassessment (Figure 1).

Over the following months, continued eruption revealed three additional tusk structures within the alveolus: a small ventral structure and two larger laterodorsal structures (Figure 2). Nine months after the initial treatment, all three structures were surgically removed under general anaesthesia. The smaller structure was thin and mobile, while the two larger structures were partially fused coronally to the primary tusk and required sectioning before extraction (Figure 3).

The primary tusk was retained because it continued to erupt normally (Figure 4). No further intervention was performed. At 24- and 36-month follow-up, the tusk showed normal morphology and function, with no recurrence of accessory structures (Figure 5).

### 3.2. Case 2

A wild-caught young African elephant bull was brought into captivity and was estimated to be 3 years old at that time. Both tusks appeared normal. Approximately 2 years later, the bull fell while playing with other elephants and fractured the left tusk. Records indicated that the fractured tusk remnants were removed under general anaesthesia. Approximately 2 years later, the left tusk began to erupt with a split appearance. It was decided to trim the tusk and maintain it at a short length (Figure 6). No pulp was exposed during trimming, which the elephant permitted while conscious.

Regular trimming consistently revealed three structures: one larger and two smaller tusks (Figure 7). Trimming continued at regular intervals until the elephant died at 31 years of age from a disease unrelated to the tusk abnormality. Because of concern regarding the infectious organism associated with his death, only a limited necropsy was performed and all tissues, including the abnormal tusk, were incinerated.

### 3.3. Case 3

A 29-year-old African elephant cow in a sanctuary setting was observed to have two tusks within the left alveolus (Figure 8). The primary tusk was larger, with a smaller, distally positioned secondary tusk. Both were much shorter than the single right tusk and grew straight ventrally without normal curvature. No historical or clinical information regarding the onset or progression of the condition was available. No intervention was performed.

### 3.4. Case 4

A 30-cm abnormal dry tusk specimen from an African elephant was available for evaluation at the School of Dentistry, University of Pretoria. No clinical history or other metadata relating to the animal were available. The specimen was composed of five differently sized tusks, all attached to one another coronally. Each individual tusk had a distinct pulp canal (Figure 9).

## 4. Results

The four cases are summarised in Table 1. Supernumerary tusks occurred in both sexes and in animals ranging from juveniles to mature adults. In all clinically documented cases, the left tusk was affected. The affected alveoli contained between two and five tusks. Clinical presentation ranged from incidental findings to behavioural abnormalities associated with tusk deformity. In the surgically managed case, selective removal of the accessory tusks while preserving the primary tusk resulted in a favourable long-term functional outcome.

## 5. Discussion

### 5.1. Aetiology

The diagnosis of supernumerary tusks describes the presence of additional teeth beyond the normal complement [12] and does not define their developmental origin.

Several theories have been proposed to explain the development of supernumerary teeth, including phylogenetic reversion (atavism), splitting of a tooth germ, localised hyperactivity of the dental lamina, hereditary influences, and trauma. These mechanisms are largely derived from observations in brachydont teeth, and their applicability to continuously growing elephant tusks requires careful consideration.

Atavism is unlikely to explain the findings in the present series. This theory proposes that supernumerary teeth represent re-expression of ancestral dental patterns [13]. However, additional tusks within a single maxillary incisor alveolus do not correspond to a known ancestral dental formula in elephants and do not represent restoration of a lost tooth position. The abnormalities described here were not additional teeth in a normal anatomical series, but multiple tusk-like structures occupying the same alveolus as the primary tusk.

Dichotomy of the tooth germ or splitting of a developing tooth bud (gemination) is another possibility [12]. Gemination results from attempted division of a single tooth germ and could theoretically produce a duplicated or bifid tusk. It does not, however, account for the multiple distinct tusks demonstrated in Cases 1 and 4. Fusion, by contrast, results from union of two separately developing tooth germs [12]. Although fusion could account for union between adjacent developing tusks, it does not explain the formation of multiple additional tusks and cannot be assessed retrospectively in the present cases. Localised hyperactivity of the dental lamina resulting in accessory tooth bud formation is a plausible mechanism for many supernumerary teeth in humans and domestic animals [12,15]. Its applicability to the present cases is uncertain. Elephant tusks are elodont teeth with lifelong growth, and the supernumerary tusks in this series appeared within the same alveolus as an established primary tusk. The available clinical evidence cannot demonstrate whether a separate, fully organised accessory tooth germ arose from dental lamina.

A hereditary basis cannot be excluded. Supernumerary teeth have been associated with several syndromes in humans, although their patterns of inheritance and development remain incompletely understood. They also occur more frequently in affected human families than in the general population [16]. Similarly, supernumerary maxillary fourth premolar teeth have been described as a heritable condition in related Burmese cats [21]. The present case series, however, provides no evidence of familial occurrence, population clustering, or recurrence in related individuals. A hereditary component has also been recognised in some supernumerary dental abnormalities in other mammalian species, but whether this applies to supernumerary tusks in elephants is unknown [16,21].

Trauma may be relevant to Case 2, but a causal relationship cannot be established. Both tusks appeared clinically normal before the fracture, and multiple tusks were recognised only during subsequent eruption after removal of the fractured tusk remnant. This temporal sequence is compatible with trauma-related disturbance of odontogenic tissues, as previously proposed [9,10], but other explanations remain possible, including a pre-existing abnormality that was not clinically apparent before the fracture, or altered exposure and eruption of pre-existing structures following the fracture and subsequent removal of the tusk remnant. Case 2 therefore demonstrates an association between trauma and the subsequent recognition of multiple tusks, rather than evidence that trauma caused their formation.

Taken together, conventional theories of supernumerary tooth formation do not fully explain the findings in this series. The continuously growing nature of elephant tusks may permit developmental or reparative processes not encountered in conventional rooted teeth. Following eruption and loss of the enamel cap, dentine production by odontoblasts lining the pulp cavity persists throughout life. It is therefore conceivable that displaced, sequestered, or ectopic odontogenic mesenchymal tissues associated with the tusk apex or periapical environment may retain the capacity to form independent dentine-producing structures. These structures may then erupt alongside the primary tusk as dentinogenesis continues.

The independently erupting, morphologically organised, and surgically separable structures in Case 1 support their interpretation as distinct teeth adjacent to a primary tusk within the same alveolus. The term “tusklet” has historically been used descriptively for such structures but may understate their biological organisation. We therefore propose that they are more appropriately regarded as supernumerary tusks (supernumerary teeth) occurring in elodont dentition.

Concrescence occurs when two separate teeth become united by cementum deposition [12]. Because concrescence presupposes the presence of two independently formed teeth, it cannot explain the formation of the supernumerary tusks. It may, however, explain the attachment observed between adjacent tusks. In Case 1, some of the supernumerary tusks were attached to the primary tusk, while in Case 4 all five tusks were united coronally. The gross appearance of these attachments and the ability to separate the structures in Case 1 without disrupting the primary tusk suggest that a process analogous to concrescence may have occurred. Although histological confirmation was unavailable, continued cementum deposition associated with these elodont teeth could have produced secondary union between adjacent tusks.

All three clinical cases involved the left tusk; however, with only three observations and the side of Case 4 unknown, it cannot be determined whether this represents a true lateral predisposition or a chance finding.

### 5.2. Differential Diagnoses

Odontomas are hamartomatous odontogenic lesions and are classified as complex or compound odontomas [22]. Compound odontomas consist of numerous small tooth-like structures. They rarely erupt and may occur in the gingiva distant from a tooth [22]. The abnormalities described in the present study showed organised morphology, continued eruption, and gross structural resemblance to normal tusks rather than the irregular mineralised architecture generally associated with odontomas.

Comparison with odontomas should nevertheless be made cautiously because existing classifications and descriptions are derived almost exclusively from brachydont dentition. Comparatively little is known about odontogenic developmental abnormalities affecting elodont teeth. Boy and Steenkamp [23] described proliferative odontogenic lesions associated with the apical regions of elodont yellow tree squirrel (*Paraxerus cepapi*) incisor teeth and proposed the term elodontoma to distinguish these lesions from odontomas arising in conventional brachydont dentition. These lesions contained enamel, dentine, and cementum associated with the apical region of the incisor tooth. Elodontomas are generally regarded as a disease of captivity and have frequently been associated with traumatic injury to incisor teeth. Radiographically, they appear as radiopaque masses associated with the tooth apex and may obstruct normal eruption [23]. Unlike the present cases, the squirrel lesions did not form independent, erupting dental structures, but consisted of disorganised odontogenic tissue associated with the apical region of the tooth. Their biological behaviour therefore differs fundamentally from the structures described here.

Based on the clinical, surgical, and gross morphological findings, the most appropriate interpretation is that these abnormalities represent supernumerary tusks associated with the continuously growing dentition of elephants.

### 5.3. Clinical Relevance

Supernumerary tusks may have important clinical implications, particularly when associated with altered tusk morphology, behavioural abnormalities, or secondary trauma. In Case 1, behavioural changes preceded treatment, suggesting that the abnormality may have caused discomfort or altered tusk function. The multiple openings in the tusk also raised concern regarding communication with the pulp cavity and the risk of ascending contamination or infection.

Diagnosis of tusk abnormalities in elephants remains challenging. Conventional radiography was non-diagnostic in Case 1 because of the density and dimensions of the tusk and surrounding alveolar bone. Clinicians may therefore need to rely primarily on clinical examination, gross morphology, and longitudinal assessment of eruption patterns when advanced imaging is unavailable or impractical.

This series demonstrates that conservative management combined with selective removal of supernumerary tusks may provide a favourable long-term outcome while preserving the primary tusk. In Case 1, the primary tusk continued to erupt and remained functional after removal of the accessory tusks, with normal morphology and wear still evident 36 months later. Preserving the primary tusk is clinically important because tusks are extensively used for feeding, digging, social interaction, environmental manipulation, and defence [1,18].

Calcium hydroxide was used as an intracavitary dressing in Case 1 to reduce bacterial contamination and promote hard-tissue formation should viable pulp tissue have been present deeper within the tusk [24]. Epoxy resin was used as a temporary protective covering to limit further contamination. Because clinically documented supernumerary tusks are rare, their long-term biological behaviour remains uncertain. Continued clinical reporting will be important to improve understanding of their development, progression, and optimal treatment.

## 6. Conclusions

This study provides the first detailed clinical description and long-term management of supernumerary tusks in African savanna elephants (*Loxodonta africana*). Historical reports have documented multiple tusks in elephants, but were largely observational and lacked clinical evaluation, surgical management, or longitudinal follow-up.

The findings support the diagnosis of these abnormalities as supernumerary tusks. Their precise developmental mechanism remains uncertain, and the continuously growing nature of elephant tusks may permit developmental or reparative processes not encountered in brachydont dentition. Trauma may contribute in at least some cases.

Selective removal of accessory tusks while preserving the primary tusk can result in a favourable long-term functional outcome. Further clinical, developmental, and histopathological investigations are required to clarify the biological basis, prevalence, and clinical significance of supernumerary tusks in elephants.

## Figures and Tables

**Figure 1:**
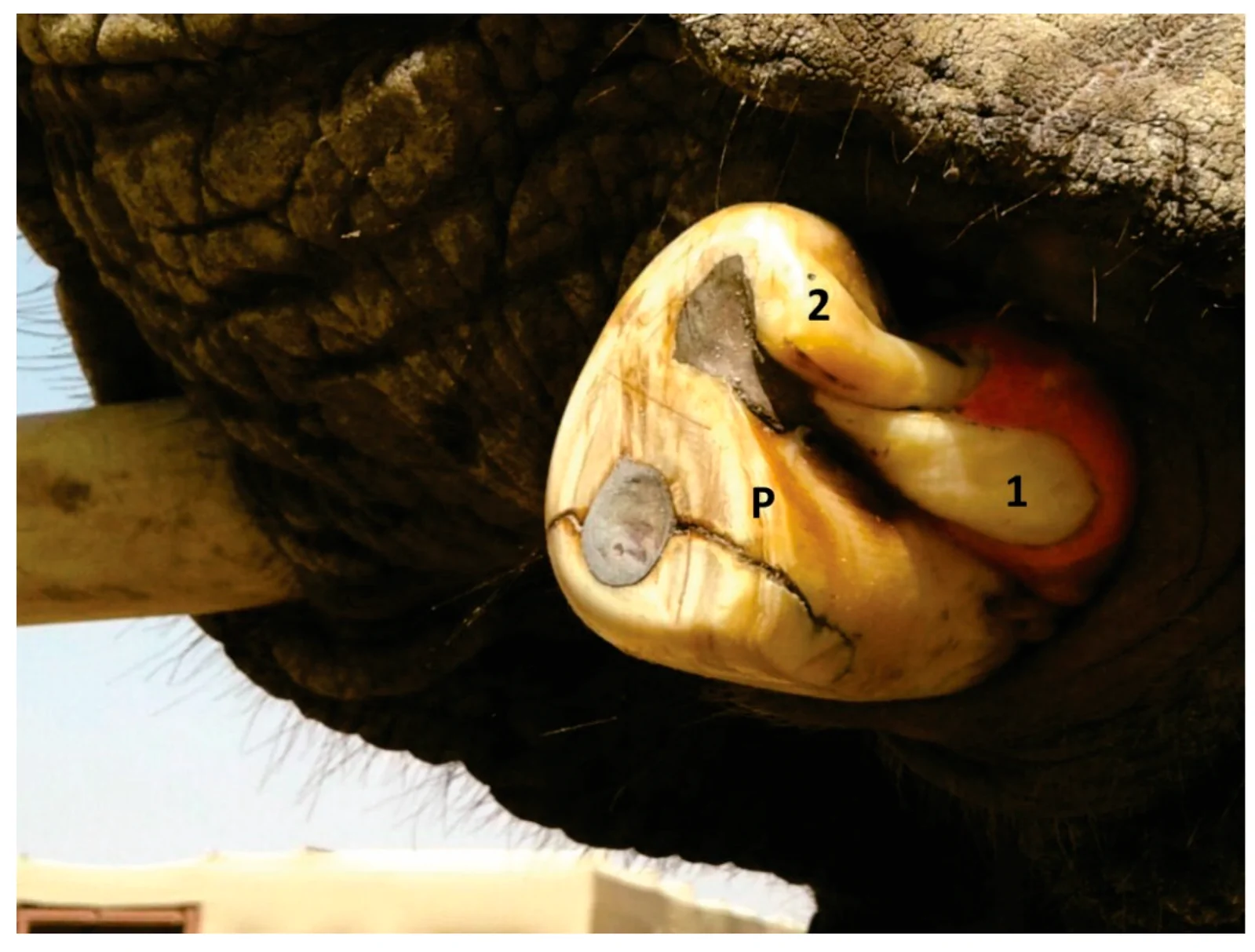
The cleaned, abnormal primary left tusk (P) of a young African elephant bull shortly after initial treatment with calcium hydroxide and a temporary epoxy resin covering. The two supernumerary tusks visible on this view are marked as 1 and 2.

**Figure 2:**
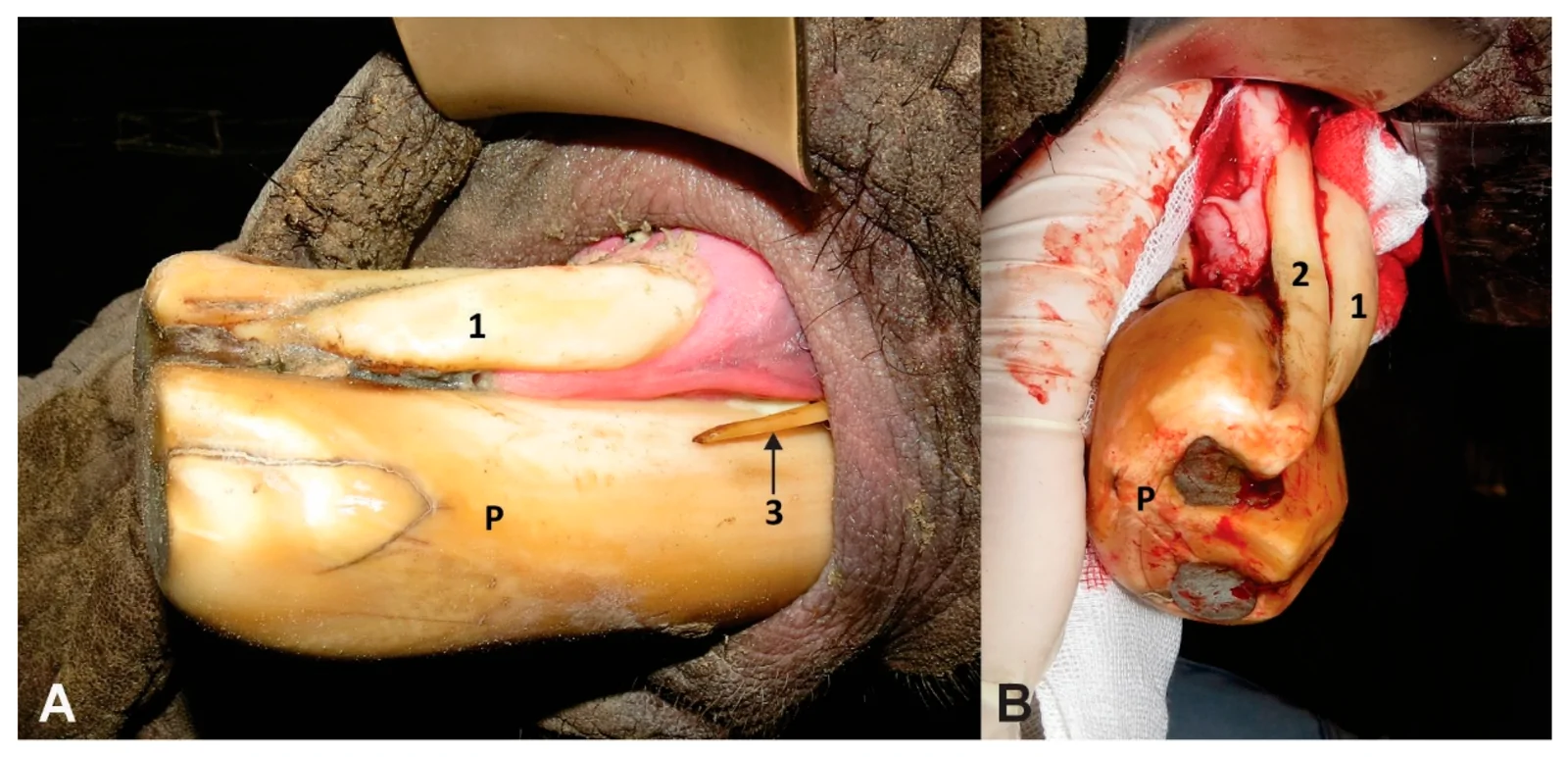
(**A**) A third supernumerary tusk (3) visible at the time of surgery. (**B**) The two larger, more laterally positioned supernumerary tusks (1 and 2) attached to the primary tusk (P).

**Figure 3:**
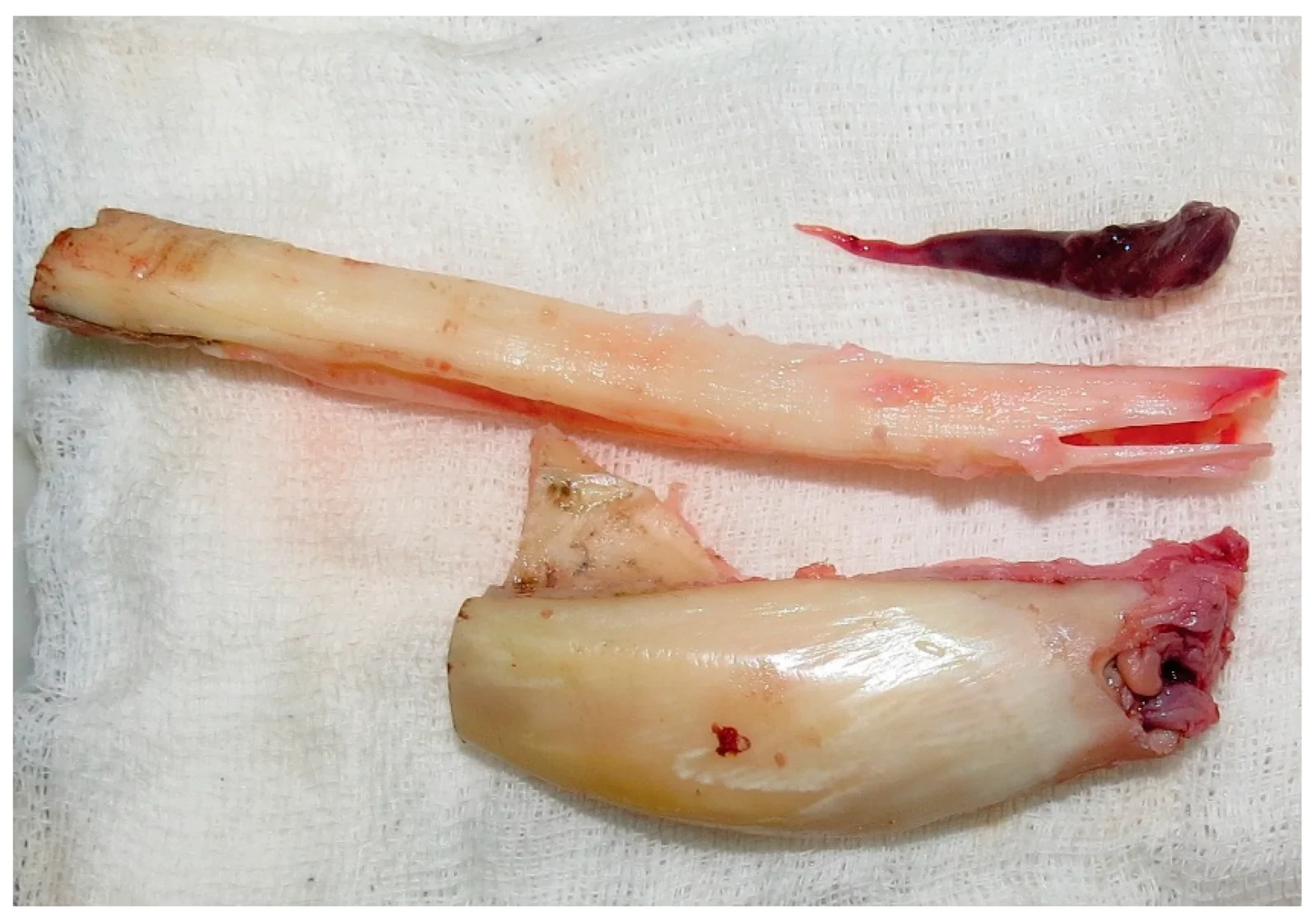
The two larger supernumerary tusks after sectioning and removal. Pulp tissue removed from the more dorsally positioned tusk is shown adjacent to it, while the lower structure retained its pulp tissue.

**Figure 4:**
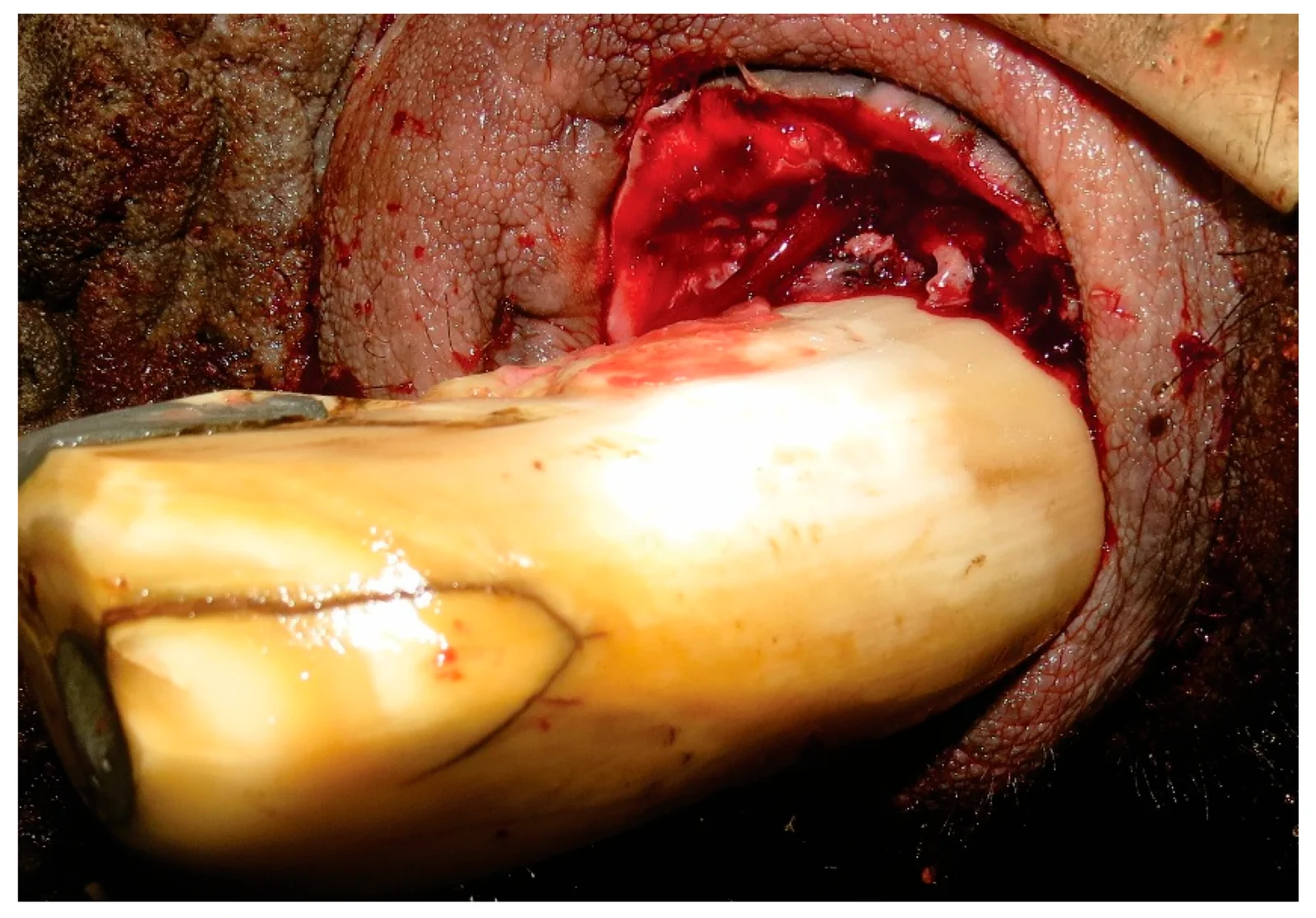
The remaining primary tusk after extraction of the three supernumerary tusks in a young African elephant bull.

**Figure 5:**
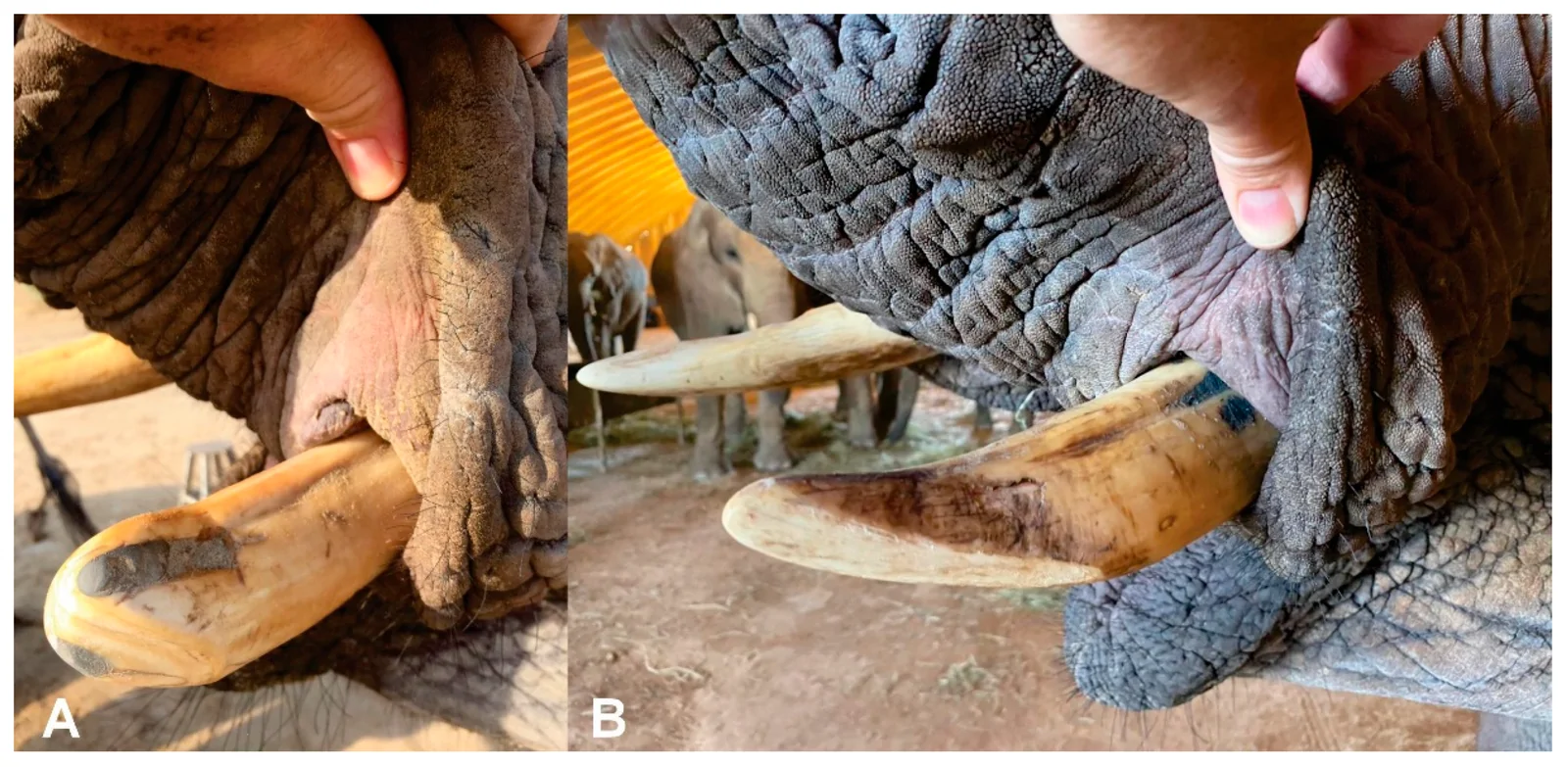
(**A**) The epoxy resin remained visible on the coronal aspect of the growing tusk 24 months after extraction of the supernumerary tusks. (**B**) At 36 months, the epoxy resin was no longer visible and the shape of the tusk approximated that of the contralateral tusk.

**Figure 6:**
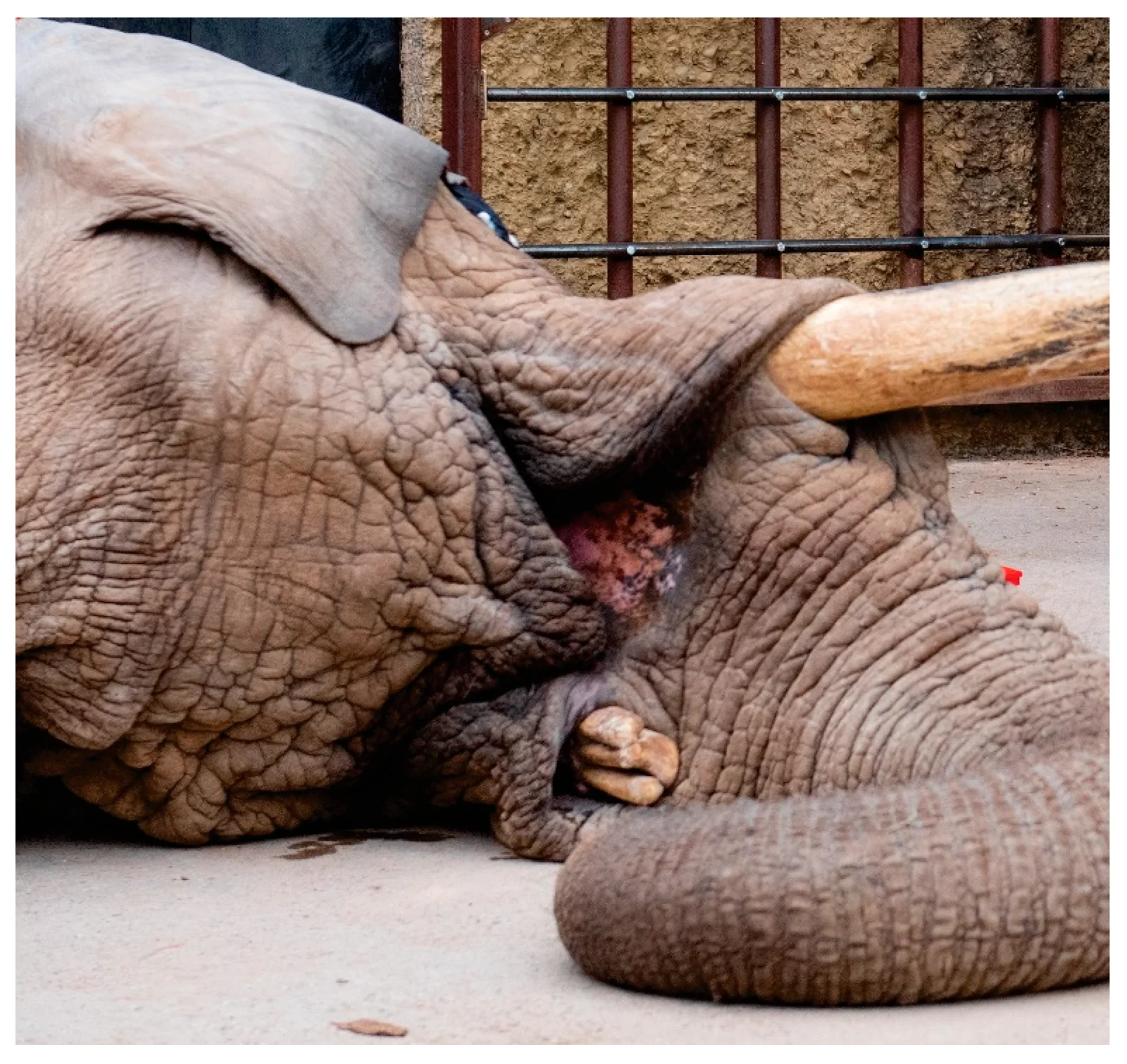
An adult African elephant bull with three separate tusks present in the left alveolus. It is difficult to determine; however the central tusk appears to be the primary tusk.

**Figure 7:**
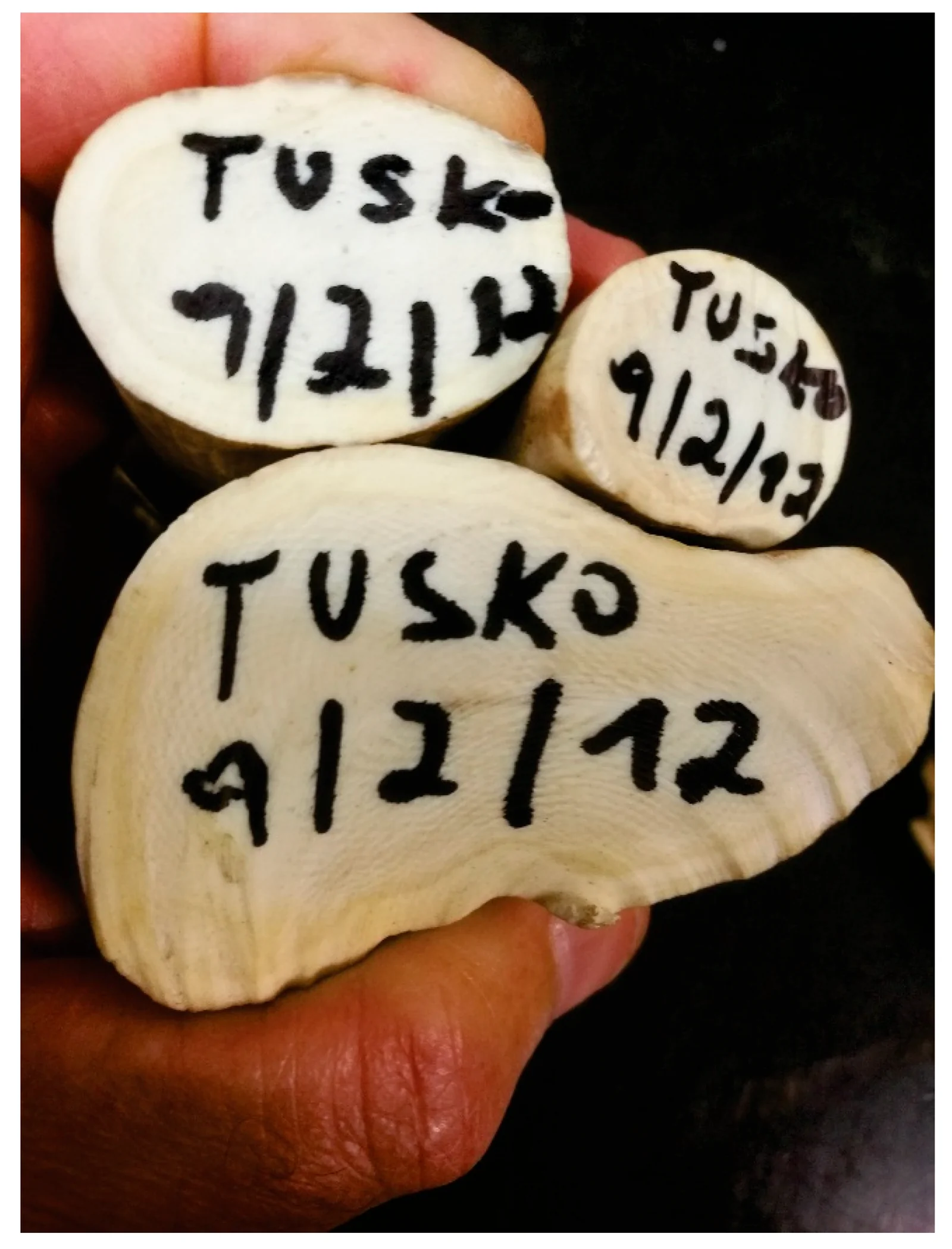
Coronal sections of the three tusks present in the left alveolus of an adult African elephant bull after trimming.

**Figure 8:**
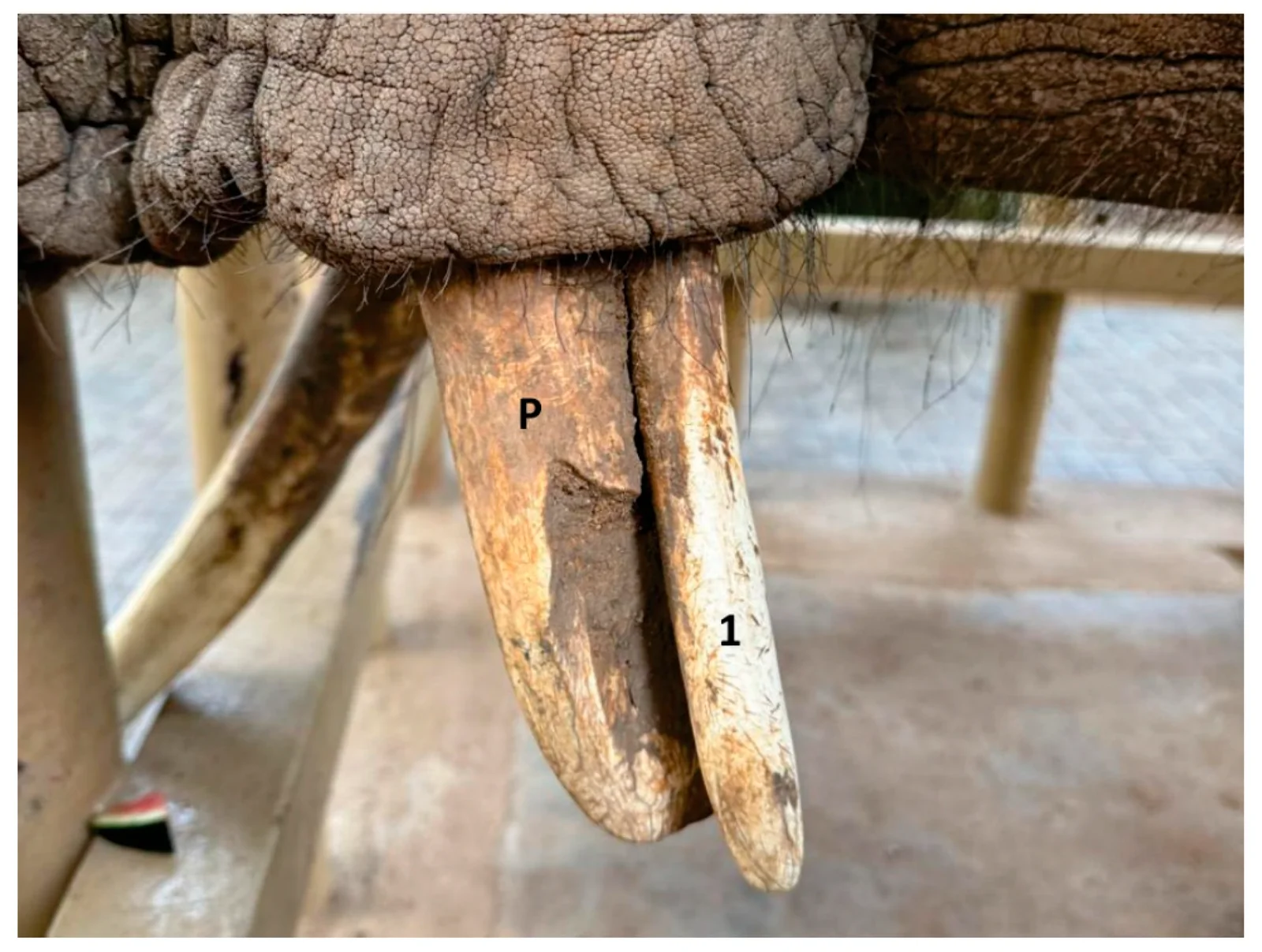
Primary left tusk (P) and supernumerary tusk (1) in an adult African elephant cow. The contralateral right tusk had normal length and curvature, whereas both left tusks erupted in a straight ventral direction and lacked the normal curvature.

**Figure 9:**
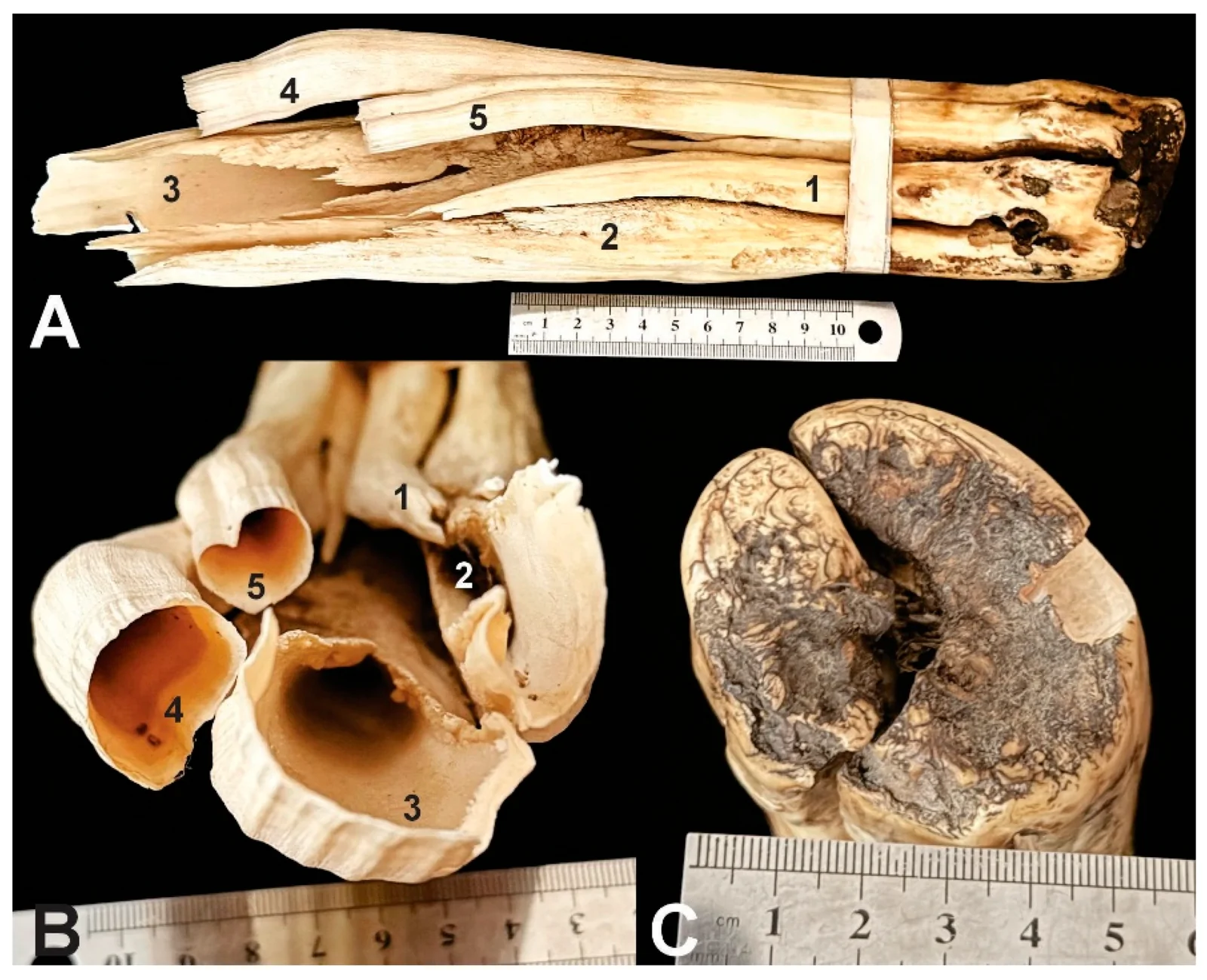
Dorsal (**A**), apical (**B**), and coronal (**C**) views of the multiple tusks from an African elephant. Five distinct pulp canals are visible apically, while the tusks were united coronally. The five tusks are numbered (1–5) for ease of reference.

**Table 1:** Summary of the four African savanna elephant cases, including age and sex, origin and setting, affected tusk and configuration, diagnostic evidence, and management and outcome. Age represents the age at first recognition of the tusk abnormality.

Case	Age (Years)	Sex	Tusk Affected		Total Tusks in Affected Alveolus	
1	7–10	Male	Left		4	
2	3–31	Male	Left		3	
3	29	Female	Left		2	
4	Unknown	Unknown	Unknown		5	
**Case**	**Age (Years) at First Recognition**	**Sex**	**Origin/Setting**	**Affected Tusks and Configuration**	**Diagnostic Evidence**	**Management Outcome**
1	7	M	Brought into captivity; captive	Left; 1 primary + 3 supernumerary tusks	Clinical examination, serial eruption and surgical findings; pulp tissue present in supernumerary tusks	Supernumerary tusks removed; primary tusk retained and functional at 36 months
2	7	M	Brought into captivity; captive	Left; 1 primary + 2 supernumerary tusks	Repeated clinical examination and trimming demonstrated three distinct tusks	Regular trimming until death at 31 yr from unrelated disease
3	29	F	Origin unknown, captive	Left; 1 primary + 1 supernumerary tusk	Gross clinical examination	No intervention
4	Unknown	Unknown	Unknown	Side unknown; 5 supernumerary tusks. None big enough to be called primary	Anatomical specimen; five distinct pulp canals	Not applicable

## Data Availability

All data presented in this study are contained within the article. Additional case information may be available from the corresponding author upon reasonable request, subject to institutional and case confidentiality.
